# Predictive factors of virological success to salvage regimens containing protease inhibitors in HIV-1 infected children

**DOI:** 10.1186/1471-2334-7-55

**Published:** 2007-06-10

**Authors:** Beatriz Larru, Carmen de Mendoza, José Ma Bellón, Ma Isabel de José, Ma José Mellado, Vincent Soriano, Ma Angeles Muñoz-Fernandez, José T Ramos

**Affiliations:** 1 Laboratorio de Inmuno-Biología Molecular, Hospital General Universitario "Gregorio Marañón", Madrid, Spain; 2 Enfermedades Infecciosas; Hospital "Carlos III", Madrid, Spain; 3 Pediatría-Infecciosas; Hospital Universitario "La Paz", Madrid, Spain; 4 Pediatría-Infecciosas; Hospital "Carlos III", Madrid, Spain; 5 Inmuno-Pediatría; Hospital Universitario "12 de Octubre", Madrid, Spain

## Abstract

**Background:**

The impact of HIV drug resistance mutations in salvage therapy has been widely investigated in adults. By contrast, data available of predictive value of resistance mutations in pediatric population is scarce.

**Methods:**

A multicenter, retrospective, observational study was conducted in children who received rescue salvage antiretroviral therapy after virologic failure. CD4 counts and viral load were determined at baseline and 6 months after rescue intervention. Genotypic HIV-1 resistance test and virtual phenotype were assessed at baseline.

**Results:**

A total of 33 children met the inclusion criteria and were included in the analysis. The median viral load (VL) and median percentage of CD4+ at baseline was 4.0 HIV-RNA log copies/ml and 23.0% respectively. The median duration that children were taking the new rescue regimen was 24.3 weeks (23.8–30.6). Overall, 47% of the 33 children achieved virological response at 24 weeks. When we compared the group of children who achieved virological response with those who did not, we found out that mean number of PI related mutations among the group of responders was 3.8 *vs*. 5.4 (p = 0.115). Moreover, the mean number of susceptible drugs according to virtual phenotype clinical cut-off for maximal virologic response was 1.7 *vs*. 0.8 and mean number of susceptible drugs according to virtual phenotype cut-off for minimal virlologic response was 2.7 *vs*. 1.3 (p < 0.01 in all cases). Eighteen children were rescued with a regimen containing a boosted-PI and virological response was significantly higher in those subjects compared with the others (61.1% *vs*. 28.6%, p < 0.01).

**Conclusion:**

Salvage treatment containing ritonavir boosted-PIs in children with virological failure was very efficient. The use of new tools as virtual phenotype could help to improve virologic success in pediatric population.

## Background

Treatment with highly active antiretroviral therapy (HAART) has resulted in great reductions in mortality and progression of HIV-1 disease in both adults and children. Increasingly, these children are surviving through to adolescent into adult life [1]. However, children taking antiretroviral therapy tend to present with higher plasma viral load (VL) and lower virologic response rates than adults. Whenever viral replication is inefficiently controlled, virologic failure happens more quickly, allowing the selection of HIV-1 quasispecies resistant to antiretroviral drugs [2,3].

Guidelines for antiretroviral treatment (ART) in children recommend an early and active approach, which usually includes one Protease Inhibitor (PI) or Non-Nucleoside Analogue Reverse Transcriptase Inhibitor (NNRTI) and two Nucleosides Analogue Reverse Transcriptase Inhibitors (NRTI) [4]. Although it includes the same antiretroviral drugs as in adults, ART in children has special features like: unsuitable formulations with inadequate pharmacokinetics and poor palatability and lack of compliance which is usually one of the main causes of lower response rates in children [5,6].

The use of co formulation of PIs with a fixed dose of ritonavir (rit) has shown a greater activity in both antiretroviral-naïve and treatment experienced HIV-1 infected children than previous PIs because of its pharmacokinetic advantages due to low-dose of rit which enhance the antiretroviral activity of the other PI. [7-9]. However, the extended use of PIs as salvage therapy for heavily pre-treated subjects has not fulfilled its expectations [10-12]. Moreover, there are few studies available concerning predictive factors of virological success when PIs/rit are used in a second or third-line of HAART in this special population [7,13].

The aim of this retrospective study was to analyze and determine predictive factors of virological success when PIs are used as a salvage therapy for HIV-1 infected children.

## Methods

### Population and study design

A multicenter retrospective, observational study was conducted to evaluate the predictive factors of virological responses when PI-containing regimens were used as salvage therapy in vertically HIV-1 infected children. Patients were recruited from three large Spanish Hospitals (Hospital Universitario Doce de Octubre, Hospital Infantil La Paz and Hospital Carlos III), with a specialized HIV/AIDS unit in pediatrics. The inclusion criteria were: (1) older than one year of age, (2) been previously treated with antiretroviral therapy including PIs, (3) at least 6 months of follow-up with a salvage regimen and (4) having a resistance test (genotypic and phenotypic) at baseline.

### Viral load and CD4 cell count measurements

Plasma viremia was determined using the bDNA assay version 3.0 (Bayer, Barcelona, Spain), which has a lower detection limit of 50 HIV-RNA copies/ml. A Virological Response (VR) was considered as significant when plasma HIV-RNA reductions were greater than 1 log and/or went to less than 50 HIV-RNA copies/ml. The CD4+ lymphocyte count was measured by flow cytometry (Coulter, Madrid, Spain). Patients were monitored every three months. Clinical examination and laboratory tests were performed at each visit. There was not a uniform approach regarding antirretroviral drugs included in the salvage regimen. Instead, each paediatrician administered the appropriate ART regimen and changed the drugs according to his/her interpretation of the children data. The adherence of antiretroviral drugs included in the salvage regimen, was measured by each paediatrician by pill count and through interviews with their parents or tutors.

### Resistance analysis

Genetic sequences of both HIV *protease *(*PRO*) and *retrotranscriptase (RT) *genes were obtained at baseline using and automatic sequencer (ABI Prism 3100, Applied Biosystems, Foster City, CA). Interpretation of drug resistance mutations was performed following the latest IAS-USA guidelines [9].

### Virtual phenotype

Genetic sequences obtained at baseline were sent to Virco for the estimation of the virtual phenotype (vPT). vPT is a probabilistic estimate of the phenotype (fold-changes in IC_50_) for a patient's HIV genotype by matching it with other genotypes available in large databases in which they are paired with phenotypes. The phenotypes matching distinct genotypes close to the one tested are used to produce an average phenotype for each drug. This strategy may allow the use of phenotypic information avoiding the difficulties and cost of real phenotypes. Results of the analysis were expressed for each drug as Clinical cut-off values (CCO) which are based on virologic response observation (change in VL) in treated patients, and give an indication of how response is affected by viral resistance. CCO provide a higher degree of correlation with virologic outcome. The lower clinical cut-off (CCO1) is the baseline Fold Change associated with 20% loss of the wild type virologic response due to viral resistance. The upper clinical cut-off (CCO2) is the baseline Fold Change with an 80% loss of the wild type virologic response due to viral resistance. CCO1 and CCO2 were considered for each drug according to the latest values described by Virco.

### Statistical analyses

Continuous and categorical data were expressed as median [interquartile ranges (IQR)] and percentages respectively. Univariate analysis was performed to compare baseline characteristics following virological response. Categorical and continuous variables, were compared with the chi-square test (Yates and Fisher correction when necessary) and non-parametric tests (Mann-Whitney U), respectively. Univariate and multivariate binary logistic regression analyses were used to identify baseline characteristics associated with VR. All statistical analyses were conducted using the SPSS package (v11.0; Chicago, IL, USA), and all differences were considered to be significant when p values were below 0.05.

## Results

A total of 33 children were included in the study. Distribution according to sex was; 17 boys (51.5%) and 16 girls (48.5%). Baseline characteristics of the study population are recorded in Table 1. The median age at baseline was 11.7 (7.5–14.2). Median time of previous exposure to antiretroviral drugs among the whole group of patients was 83 months (49–119) and by drug families was 84 months (49–119) for NRTIs, 5 months (0.0–31) for NNRTIs and 61 months (38–73) for PIs.

**Table 1:** Baseline characteristics of the study population.

N	33
Sex (%)	
Male	51.5
Median Age (years)	11.7 (7.5–14.2)
Median % of Baseline CD4 counts	23.0 (19.5–32.0)
Median % of Baseline CD8 counts (%)	45.0 (33.5–57.5)
Median Baseline VL (HIV-RNA Log copies/ml)	4.0 (3.4–4.6)
Length of ARV therapy (months)	83.0 (49.0–119.0)
Length of PI exposure (months)	61.0 (38.0–73.0)
Length of NRTI exposure (months)	84.0 (49.0–119.0)
Length of NNRTI exposure (months)	5.0 (0.0–31.0)
Median number of previous PI exposure	2.0 (1–3.0)
Median number of NAMs	3.0 (1.5–4.0)
Median number of protease resistance mutations	5.0 (2.0–6.5)

Results are expressed as median (IQR) (VL: viral load; ARV: antirretroviral treatment; PI: protease inhibitors; NAMs; nucleotide analogue mutations: M41L, D67N, K70R, L210W, T215Y/F y K219Q/E/R/S/N)

All children had been exposed to at least one PI before the first determination. The proportion of children who have been treated with different PI were as follows: Nelfinavir (NFV) (78.8%), Lopinavir/ritonavir (LPV/rit) (36.4%), Ritonavir (RTV) (30.3%), Indinavir (IDV) (21.2%), Saquinavir (SQV) (21.2%) and Amprenavir (APV) (6.1%). The number of PI that the children have taken before the baseline determination was: 48.6% had taken 1, 24.2% had taken 2, 15.1% had taken 3, 9.1% had taken 4 and 3% had taken 5. At baseline all the children take a PI as part of the salvage regimen. At time of the analysis, 60.6% were using NFV, 30.3% LPV/rit, 6.1% IDV and 3.0% SQV/rit.

The median CD4+ cell counts and percentage at baseline were 707.0 (537.5–941) and 23.0 % (19.5–32), and the CD8+ cell counts was 1309.0 (967.2–1996.5) and 45.0% (33.5–57.5). Median baseline viral load was 4.0 (3.4–4.6) HIV-RNA log copies/ml. At baseline, the median number of Nucleotide Analogue Mutations (NAMs) was 3.0 (1.5–4) and the median number of protease resistance mutations was 5.0 (2–6.5). M184V was found in 11 children (33%); 3 of them (30.3%) were resistant to Abacavir and 1 of them (15.2%) was resistant to Tenofovir according to the virtual phenotype. At baseline, 42.4% of children have NNRTI mutations; 30.3% have the K103N and 15.2% the Y181C.

Virological response defined as plasma HIV-RNA reductions greater than 1 log and/or VL less than 50 HIV-RNA copies/ml was achieved in 46.9% of children in the first 24 weeks of follow-up. However, 66.6% of them reached undetectable viremia after 24 weeks of follow-up. When we analyze the decrease in VL among patients who achieved virologic response and those who did not, we found the following differences: the former group had a mean decrease of log VL of -1.8 *vs*. -0.2 (p = 0.01). Furthermore, when we compared the variation of the percentage of CD4+ and CD8+ cell counts between the two groups, virological responders had a mean increase in the %CD4+ cell count of 5.3 *vs*. 0.6 (p = 0.14) and a mean decrease of %CD8+ cell count of -4.7 *vs*. 0.6 (p = 0.01). Finally, when we compared the group of children who achieved virological response with those who did not, we found the following differences: mean number of PI related mutations was 3.8 *vs*. 5.4 (p = 0.11), mean number of susceptible drugs according to cut-off for maximal virologic response (CCO1) 1.7 *vs*. 0.8 (p = 0.03) and mean number of susceptible drugs according to cut-off for minimal virlologic response (CCO2) 2.7 *vs*. 1.3 (p = 0.01), respectively.

PI combinations with low doses of ritonavir were used in 18 children; 15 received LPV/rit, 2 Fosamprenavir/rit and 1 SQV/rit. None of them received a double boosting of PIs. Virological response was significantly higher in those subjects rescued with boosting PIs combinations compared with the others (61.1% *vs*. 28.6%, p = 0.09).

In the univariate analyses, there was no association between the number of baseline protease resistance mutations and virological response. Moreover, there was also not association between virological success and RT resistance mutations, the previous length of antiretroviral therapy, previous length of PI exposure, number of PI previously received or rescue interventions with boosted-PI. Nevertheless, we found association between the number of drugs considered as susceptible according to CCO1 and CCO2 and the use of an active PI in salvage regimens (Table 2). In the multivariate analysis, a lower number of PI previously received and a higher number of drugs considered as susceptible according to CCO2 were independently associated with virological response (Table 2).

**Table 2:** Factors associated with virological response (univariate and multivariate analyses).

	**Univariate analysis**		**Multivariate analysis**	
	**OR (95% CI)**	**p**	**OR (95% CI)**	

Rescue with PI/rit	3.93 (0.88 to 17.56)	0.07	7.57 (0.58 to 99.31)	0.12
Previous length of ARV exposure	0.99 (0.97 to 1.01)	0.29	-	-
Previous length of PI exposure	0.98 (0.94 to 1.01)	0.19	-	-
No. previous PIs exposure	0.61 (0.29 to 1.25)	0.18	0.23 (0.06 to 0.89)	**0.03**
No. susceptible drugs according to CCO1	2.16 (1.02 to 4.57)	**0.04**	0.22 (0.03 to 1.57)	0.13
No. susceptible drugs according to CCO2	4.99 (1.55 to 16.05)	**0.01**	5.34 (1.23 to 23.11)	**0.02**
No. active PIs in the salvage regimen according to virtual phenotype	6.60 (1.40 to 31.05)	**0.02**	0.64 (0.03 to 11.58)	0.76
No. NAMs	0.85 (0.54 to 1.32)	0.46	-	-
No. protease resistance mutations	0.81 (0.62 to 1.06)	0.12	0.89 (0.56 to 1.46)	0.65

(OR: Odds ratio; PI: protease inhibitor; RIT: ritonavir; ARV: antiretroviral treatment; CC01: lower clinical cut-off; CC02: upper clinical cut-off; NAMs: nucleotide analogue mutations: M41L, D67N, K70R, L210W, T215Y/F y K219Q/E/R/S/N)

## Discussion

In this study, we evaluate the predictive factors of success when PIs are used as salvage therapy in a group of heavily pretreated HIV-infected children with virological failure. We found that nearly half of them achieve virological response after rescue interventions. Our result are similar to other studies which also have been carried among children in real life situations [8,12].

Several factors were associated with virological response and could help to predict success. The numbers of drugs susceptible defined by the clinical cut-off of the virtual phenotype, as well as the use of an active PI in the rescue intervention were significantly associated with virological response in the univariate analyses. Moreover, rescue intervention based in boosting-PI combination, a lower number of baseline protease resistance mutations and a lower number of previous PI exposure were also associated with virological response although it not reach statistical significance. It was probably due to the small size of the study population. These factors appear to be significantly associated with virological response in rescue intervention in studies conducted in adult population [7,10]. The presence of 5 more protease resistance mutations as well as the use of boosting-PI combinations has been associated with significant virological response in adults who initiated rescue intervention based on boosting-PI combinations. However, children usually have lower virologic response rates than adults due partially to lower treatment adherence leading to underestimation in the factors associated with virological response. Besides, the fact that viral loads are higher among children than in adults, can lead to the development of resistance mutations which could reduce the effectiveness of antiretroviral drugs in further combinations.

Our study has several limitations primarily due to the small sample size. We did not asses the relevance of other classes of antiretroviral drugs in the rescue regimen because HAART combinations were not homogeneous in the population as it is a retrospective study based in a real life situation. In addition, some variables identified as predictive factors, like number of active PIs in salvage regimen and number of drugs considered as susceptible according to virtual phenotype might not be independent. Moreover, we did not measure other factors such as pharmacokinetic or drug interactions which might have had influence in the virological success of our population. Besides, global adherence in our cohort was around 90% (data not shown) but it was not identify as a prognostic factor due to the short period of follow-up included in our study.

In the multivariate analyses only the number of drugs used according with the CCO2 and a lower number of previous PI received were independently associated with virological response. Clinical cut-offs are based on virological response observations in treated patients giving an indication of how the response is affected by viral resistance [3,14]. This approaching provide a higher degree of correlation with virologic response in a given patient [7,15-18]. CCO2 shows the 80% loss of the wild type virologic response due to resistance. That means resistance patterns that compromise partially the effectiveness of the drug. In our study, the inclusion of drugs considered as partially susceptible measured by the CCO2 provided a favourable virologic response. In children with multiple exposures to different antiretrovirals and who have developed complex resistance patterns, the identification of drugs partially active is useful for the design of rescue intervention in this population. However, more studies with higher sample sizes which evaluate the usefulness of new tools like virtual phenotypic resistance test or the combination of pharmacokinetics parameters and resistance test are needed to improve the management of HIV-infected children.

## Conclusion

In conclusion, our results showed higher rates of virological success when *boosted*-PIs are included in salvage regimens. New strategies as virtual phenotype could improve virological responses and could contribute to select the most appropriate regimen in treatment experienced children, when antiretroviral drugs are not so available.

## Competing interests

The author(s) declare that they have no competing interests.

## Authors' contributions

BL had primary responsibility for protocol development, collecting and recording data, and outcome assessment and contributed to the writing of the manuscript. CM contributed to the writing of the manuscript and developed the preliminary data analysis. JM Bellón participated in analytic framework for the study. VS contributed to the writing of the manuscript. Paediatricians: MIJ, MJM and JTR were responsible for patient screening, and contributed to the writing of the manuscript. JTR supervised the design and execution of the study, the final data analyses, and the writing of the manuscript. All authors read and approved the final manuscript

## Pre-publication history

The pre-publication history for this paper can be accessed here:


